# Constructing Well-Being and Poverty Dimensions on Political Grounds

**DOI:** 10.1007/s11205-017-1618-0

**Published:** 2017-04-04

**Authors:** Francesco Burchi, Pasquale De Muro, Eszter Kollar

**Affiliations:** 1 German Development Institute, Department “Sustainable Economic and Social Development”, Bonn, Germany; 2 Department of Economics, Roma Tre University, Rome, Italy; 3 Chair of International Political Theory, Normative Orders Excellence Cluster, Goethe University Frankfurt, Frankfurt, Germany

**Keywords:** Well-being, Poverty measurement, Capabilities, Political constructivism, Constitutions, Italy

## Abstract

The paper addresses the problem of justifying ethically sound dimensions of poverty or well-being for use in a multidimensional framework. We combine Sen’s capability approach and Rawls’ method of political constructivism and argue that the constitution and its interpretative practice can serve as an ethically suitable informational basis for selecting dimensions, under certain conditions. We illustrate our Constitutional Approach by deriving a set of well-being dimensions from an analysis of the Italian Constitution. We argue that this method is both an improvement on those used in the existing literature from the ethical point of view, and has a strong potential for providing the ethical basis of a conception of well-being for the public affairs of a pluralist society. In the final part, we elaborate on the implications for measuring well-being based on data, by ranking Italian regions in terms of well-being, and pointing out the differences in results produced by different methods.

## Introduction

Poverty and well-being are increasingly viewed as multidimensional phenomena, which cannot be captured by one single component or variable such as income or wealth. In order to measure them in a multidimensional setting, a researcher needs to address a series of questions (Alkire 2008; Nardo et al. 2008; Burchi and De Muro 2016). These are:

1. Identification of the conceptual framework
2. Selection of relevant dimensions
3. Weighting of the dimensions
4. Selection of the indicators
5. Possible aggregation of the dimensions into a composite index (if a single number is required).

In the case of poverty, an additional step before aggregation is the “identification” of the poor—i.e., setting the poverty line for each selected indicator.

In this paper, we endorse one specific multidimensional approach: Amartya Sen’s capability approach. The capability approach views well-being as made of people’s *functionings*, namely what they achieve in their life (Sen 1985a). Poverty, in this framework, is seen as “deprivation of basic capabilities”, that is, deprivation of the freedom to achieve a minimum level of basic functionings (Sen 1999). Poverty concerns this minimum level of basic or central human functions.^1^ This means that the identification of a number of basic functionings, i.e. basic well-being dimensions, that every citizen should be able to achieve corresponds to the identification of poverty dimensions.

A further complication within the ethical framework of the capability approach is that it combines an objective/social and a subjective/individual evaluation of a life worth living. As Sen writes, it “sees persons from two different perspectives: well-being and agency” and “each aspect … yields a corresponding notion of freedom” (Sen 1985b, p. 169). *Well*-*being freedom* is the freedom to achieve well-being. By contrast, *agency freedom* concerns the freedom to achieve one’s goals, which may go beyond personal well-being. The two perspectives are distinct but interrelated. Another important distinction is between “well-being achievement”, i.e. what a person actually managed to do and to be, and the above cited “well-being freedom”, which represents all potential achievements (Sen 1993). Our analysis is mostly concerned with the *well*-*being freedoms* explicitly or implicitly granted in the Italian Constitution.

There are two main comparative advantages of the capability approach as conceptual framework for poverty and well-being measurement and assessment, compared to other resource-based, multidimensional approaches. First, it can account for attributes for which a market does not exist, such as political participation or social relations. Second, by focusing on people’s capabilities and functionings, it recognizes that people’s life constraints are to be found not only in the lack of sufficient resources, but also in other factors, such as personal disability or a discriminatory labour market. The “conversion” of income or commodities into capabilities is, in fact, mediated by individual factors (e.g. age, gender, metabolism), social factors (e.g. law, social norms, power relations), and environmental factors (Sen 1985a).

Having identified the capability approach as the conceptual framework (step 1), in this paper we focus on step 2, the problem of how to select dimensions. However, it is often hard to separate the five steps from each other. A method of choosing basic well-being or poverty dimensions (step 2) might help to assign weight to each dimension (step 3) and in some cases can be used to select dimensional indicators (step 4).

We find that the problem of dimension selection is rarely considered with due care in the measurement exercises, and is rarely rooted in a coherent ethical framework. Economists and applied social scientists working in this field need guidance from philosophical theories of well-being and poverty, and justification in order to address these important questions.

We argue that Rawls’ (1993) method of political constructivism provides the ethical basis for justifying the use of the Constitution and its public culture for selecting dimensions. We define “Constitutional Approach” the specific application of Rawls’ political constructivism in this field. Then, we apply this approach to the Italian case in an attempt to derive a set of publicly justifiable dimensions of poverty and well-being. It is a long-standing Constitution with a broad political consultation at its base, which still enjoys a wide consensus. We seek to show why there is a need for more ethically sound methodological approaches to measuring poverty and well-being, pointing out the advantages of the Constitutional Approach, and how it may enrich the work of practitioners engaged in implementing poverty eradication policies.

The paper is divided into five sections. In the next section, we review the existing approaches used to derive dimensions of well-being, poverty and quality of life. Then, we explain why the political construction of poverty and well-being dimensions could be suitable for pluralist societies. In the following section, we present the example of the Italian Constitution and make an attempt to identify possible dimensions for Italy with a firm ethical foundation. In order to show the importance of selecting dimensions for policy-making, we then compare the well-being levels of Italian regions obtained using our Constitutional Approach and other methods.

## The Problem of Selecting Dimensions

How should researchers identify relevant well-being or poverty dimensions for measurement purposes? Amartya Sen (2004) points to the crucial role of deliberative processes for the identification of a shared list of valuable capabilities. The list is context-dependent as well as objective-dependent: depending on whether we want to evaluate a project, to assess poverty, to compare countries or to focus on a community, the list might be different. A key point is that the researchers should make this choice explicit. Unfortunately, most of the empirical studies on multidimensional well-being and poverty, including those rooted in the capability approach, have often failed to explain why they chose certain dimensions and which selection criteria they used (Burchi and Gnesi 2016). We argue that the selection of dimensions is a crucial step in understanding poverty in a society and to develop measures and tailor policies, and, as such, must pass the test of moral scrutiny.

An attempt to classify the existing empirical studies on multidimensional poverty on the basis of the methods employed to select dimensions has been made by Sabina Alkire (2008) in a comprehensive review article.^2^ Five different approaches were identified:

1. Data-driven approach. Here, the existence of data forms the only guiding criterion. Often, scholars do not engage in a debate on relevant dimensions, but just single out dimensions and variables that refer to some intuitive ideas of the phenomenon analyzed and for which data are available.
2. Approach founded on normative assumptions. Examples are Maslow’s (1948) pyramid of needs, as well as Nussbaum’s (2000) list of ten central capabilities, given that she builds on the Aristotelian idea of a “good life” to determine what is of core value for a dignified human life.
3. Approach based on public consensus. A list can be the outcome of “some arguably legitimate consensus building process at one point in time, and are relatively stable, thus not expected to be iterative or subject to ongoing participatory evaluation” (Alkire 2008, p. 10). Examples are the Millennium Development Goals and the Universal Declaration of Human Rights.
4. Participatory approach. Through focus groups, group discussions and other participatory techniques it is possible to draw out people’s considered values. This method can be mixed, for example, with normative assumptions, based on which the researcher identifies an initial list as a starting point for a participatory exercise (e.g. Biggeri and Libànora 2011).
5. Approach based on empirical analyses. Some scholars rely on empirical surveys, such as the World Value Survey, which show the values that are cross-culturally endorsed. Another interesting source is the recent cross-country survey “My World”, carried out as a preparation for the consultation on the Sustainable Development Goals (United Nations Development Group, 2013). This survey has been used for the identification of poverty dimensions in the work of Burchi et al. (2015).

The present paper engages in this debate, proposing a new method of justification for selecting dimensions: a revised version of the public consensus view. While Sen’s open-ended list has been criticized for not providing a specific enough account of the good life, Nussbaum’s solution to complete the idea has been criticized for relying on a too thick and singular conception of the good life. Some authors have questioned whether the capability approach to the quality of life can be reconciled with the spirit of pluralism at all (Qizilbash^1998^). By contrast, we argue that the capability approach can be reconciled with pluralism by justifying well-being dimensions on the basis of a *thin public* conception of the good. We employ Rawls’ method of political constructivism and justify well-being dimensions on grounds of public ideals embedded in the constitution and the public culture of a society. Moreover, we argue that the conservatism or status-quo bias of the public consensus view can be corrected through moral interpretation, a central normative feature of political constructivism.

## Method of Justification

In this section, we argue that political constructivism, Rawls’ (1993) method of justifying political principles for ongoing social practices, is a yet unexplored way of reasoning about well-being and poverty dimensions in the framework of the capability approach. After a brief description of the method of political constructivism,^3^ we discuss some reasons supporting this approach, and how it improves and departs from other approaches to selecting dimensions.

The basic idea of political constructivism is that political principles can be formulated by interpreting and elaborating ideas embedded in a public political culture. Therefore, the standard we use to make ethical evaluations about an institutional practice is worked out from within the practice. It is important to emphasize, first, that the object of interpretation is the *public* culture of a society. It does not, therefore, take culturally embedded conventional views or the current majority opinion to be a good starting point for ethical reasoning. Rather, it takes long-standing, crystallized and institutionally embodied ideals and norms, such as “the constitution and its public culture of interpretation” (Rawls 1993, pp. 13–14) to be the object of interpretation. The second point to stress is that the interpretation of embedded norms is not a purely descriptive exercise, but a *critical* one, aimed at political reform. It starts by assembling the key elements needed to understand what the concrete practice is about. This is the political aspect of the method. Then those elements need to be organized into a coherent whole. So the constructivist method proceeds by taking a critical stance, examining how a practice should be normatively reordered through an interpretative lens that is guided by moral theory (Dworkin 1986; James 2005; Sangiovanni 2007).

The first reason for selecting Rawls’ method stems from the need to work out public standards under the constraint of ethical pluralism. People genuinely disagree about what is fundamentally valuable in human life. Yet, many live in severe deprivation, under harsh conditions that are in urgent need of public action. The persistent philosophical disagreement about human well-being limits the potential of particular philosophical views (i.e. the purely normative approach) to generate public consensus and provide a solid foundation for public action. We think this is one important reason to move away from philosophical justifications based on moral premises referred to as the “normative approach” in the dimensions selection literature. The Rawlsian approach, by contrast, is a practice-sensitive approach to justification, which formulates political principles by reference to some central ideas embedded in its institutional framework. The ethical standard used for evaluating a practice is worked out from within, by reference to public ideas that all members participating in the institutional scheme and complying with its fundamental principles have reason to accept.

The second is a moral reason that underpins public justification; namely, respect for persons. Respect for persons as autonomous agents requires that the norms governing our shared institutions and the values that guide policies are justifiable to them. When we submit people to norms that cannot be justified to them (or they have reason to reject) we fail to respect them as persons. Purely philosophical justification grounded in a particular moral tradition fails in this basic requirement. When offered in support of political norms it fails to respect persons holding conflicting moral views. Only norms that can be endorsed from a variety of value systems satisfy this requirement in the political domain. So, we need to bracket deeper personal disagreements about value and identify public values that all citizens have reason to care about. Political constructivism holds that the values embedded in the political culture are the best candidates for that and a good starting point for justification that satisfies the respect-for-persons requirement. Then we proceed by articulating them in their best light, guided by ethical reasoning.

Third, our Rawlsian approach can avoid the charge of conservatism or conventionalism. To show this, we need to stress how our view departs from a public consensus view. We argue that the public consensus view is more conservative because it takes at face value the norms embedded in institutions. By contrast, in our political constructivist approach, institutionally embedded norms and ideas are not taken at face value, but they are placed under critical interpretation guided by moral theory. In the case below, we start by taking stock of the fundamental norms enshrined in the Italian constitution, followed by a critical interpretation through the lens of the capability approach. In other words, we take a multidimensional view of well-being and poverty that takes the enhancement of human freedom to be its central moral concern, and put that moral ideal to use in our critical interpretation of constitutional principles. This way, we achieve a critical distance from the historical product referred to as public consensus. We can take a critical stance on how institutions and policies are actually designed here and now, and press for reforming them through ethically guidance. The dimensions of well-being we get as a result start from the actual institutional consensus, but take us a step closer towards a moral ideal, thereby balancing the need for legitimacy and ethical improvement of our measures of assessment that underpin our current policies. In this sense, our approach may be seen as an ethically improved public consensus view. It is neither conservative, nor progressive. Rather, it is best seen as a reformist stance.

A final point to consider is whether all kinds of practices and any constitution would count as a good starting point for constructing a political conception of well-being. We refer to ideas embedded in a constitution in order to treat citizens as autonomous agents and equal members of a political society to whom reasonable justification is owed. However, what if those ideas are morally dubious or do not meet certain moral standards we do not want to sacrifice? Would we accept any value as a starting point just because it is embedded in a constitution? We think not. We cannot capitulate to every publicly embedded value just because it is publicly embedded. Rather, for a constitution to be a good source of ethical reasoning, it needs to meet certain criteria which we cannot fully elaborate on, let alone settle long-standing philosophical disputes about it. Without providing a conclusive answer, what we can do here is to provide some examples. We need to delineate normative threshold of a “good enough” constitutional practice as a suitable starting point. This will involve both procedural and substantive considerations. Among the procedural criteria, we need to examine the process that gave birth to the constitution, how open and inclusive it was, the extent and quality of public participation and the way diverging views have been taken into account. The public basis of support would have to be “broad enough.” Concerning the substantive criteria, we are assessing the content of constitutional norms to see if they meet the basic standards of equal concern for and treatment of persons, and respect for their relevant freedoms. Ronald Dworkin, for example, elaborates such criteria of legitimacy by reasoning about political rule that “respects the dignity of those in its power”; namely, when it shows “equal concern for the fates of all those it governs and full respect for their personal responsibility for their own lives” (Dworkin 2011, p. 352). We stress, that these threshold criteria are philosophically contested and subject of a future research program. It should suffice here that we start with cases, such as the Italian constitution that clearly meet various criteria of legitimacy, and leave aside, for the time being, more contested cases.

We argue that our political constructivist method of reasoning is a yet unexplored middle ground between Nussbaum’s moral philosophical justification of her central human capabilities and Sen’s use of public reason in locally contextualized deliberation. Sen doubts that philosophers and experts are best positioned to outline a list of basic human capabilities, and suggests that we leave the determination of basic capabilities to the process of public deliberation (Sen 2004). Nussbaum, by contrast, elaborates a list of ten central human capabilities, which she takes to be universal moral entitlements. The criterion that picks out the relevant capabilities is a dignified human life i.e. those capabilities necessary for a ‘truly human life’. She develops this idea through systematic moral reasoning based, in part, on ancient philosophical texts and ideas of the good life (Nussbaum 1990). She then presents the list as a free-standing political ideal capable of being the focus of an overlapping consensus across cultures (Nussbaum 2006). Political constructivism importantly departs from Nussbaum in its assumptions and method of justification. Nussbaum begins from observations about human nature, while our approach starts from constitutional principles. Nussbaum derives political entitlements from ethical considerations about human beings, by offering her ethical findings for public endorsement in her move to an overlapping consensus.^4^ The starting point of our approach, instead, are the institutionally embedded, long-standing political ideals that express the will of the people, and is procedurally tied to people’s sovereignty. This last point also signals our main departure from Sen. We concord with Sen that normative authority is vested in the political community; hence our insistence on starting from publicly affirmed political values. However, in order to avoid status-quo bias, in political constructivism, there is room for the role of the philosopher–theorist to interpret public ideals in a better light and push for progress.

We hold that our method is comparatively better than the ones current practices rely on, or the ones that have been proposed in the literature. In contrast to the data-driven approach, it is clear that having any method is better than not having one at all. Relying on the availability of data without any conception of well-being driving measurement and policies is in obvious tension with the normative purpose endorsed here. The normative approach is at odds with ethical pluralism (Qizilbash 1998) and it is also problematic in its failure to offer justificatory respect to citizens.

An approach that is motivated by a similar dissatisfaction with existing approaches was developed by Vizard and Buchardt (Vizard 2007; Vizard and Buchardt 2011). Vizard (2007) argues that in order to identify a list of dimensions it is necessary to integrate the capability approach with other theories of “social value.” She develops a list based on the international human rights doctrine, which, she claims, is “authoritatively recognized”, “legally significant” and capability-based. Later, Burchardt and Vizard (2011) complement this human rights approach with a second step: a participatory consultation to modify, extend and contextualize the list, for example in the United Kingdom. Their methodological commitment tips towards the human rights based list. They often stress the limitations to operationalize participatory processes, and argue that in case of conflicts, the human rights list should prevail.

There are both commonalities and departures in our political constructivist approach. One commonality is that we also stress the role of international treaties and the UDHR in the interpretation of the Italian Constitution. However, for us, the treaties may *supplement* an incomplete constitution but they do not serve as the *starting point*. By contrast, for Burchardt and Vizard the human rights doctrine is the starting point, which may be possibly supplemented with national consultations.

To point out the main normative difference in the two methods, we argue that Vizard’s human rights approach (step one) amounts to an international application of the public consensus approach. Taking the human rights enshrined in international legal documents as the basis of a list for the global community, would be a similar move to taking the constitutional principles as the basis of the list for a nation state. However, as we emphasized, if a normative argument stops at this point, it introduces a strong status quo bias or institutional conservatism. Our approach would hold, if we wanted to apply it internationally, that the list of human rights, as they are enshrined in international documents, would have to be critically examined through a complex philosophical inquiry examining the nature, foundation, and content of human rights (e.g. Sangiovanni 2007). To stress again the methodological difference, we start with a public consensus and introduce moral evaluation in the next interpretive phase. Burchardt and Vizard start with a public consensus, draw up the list, and test its local applicability in a contextualized public deliberation.

Rawls’ method of ethical reasoning, as we show in the following section, can be fruitfully employed in a justificatory argument for a political conception of well-being. In other words, when selecting dimensions that are to provide the normative material for measuring well-being and poverty in context, reasoning about the relevant dimensions should start from core ideas embedded in a public political culture. Like Rawls, we propose the constitution and its interpretative practice as the source of implicitly shared ideas in a political community, and work from there towards a publicly shareable notion of well-being. Resting the justification of well-being dimensions on political grounds, and avoiding any reference to comprehensive moral views, the hope is that the resulting public standard could be widely endorsed, despite reasonable disagreement about the good life. Such a political conception could, then, serve as the basis for public reasoning about policy goals and for measuring well-being and poverty.^5^ The idea is that selecting dimensions of capabilities in such a way could serve as a reasonable public basis of justification that could guide the measurement and policy of well-being in Italy, and could enrich our understanding of well-being and poverty more generally.

## A Case Study: The Italian Constitution

After the end of World War II, a “Constituent Assembly” was appointed in Italy by 89% of voters, with the mandate to write the national Constitution. This Assembly was representative of all the diverse political cultures (Christian, socialist, communist, conservative, liberal, republican, etc.). After a 2-years process and a large and vivid political debate, where, as expected, cultural conflicts and strong divergences on specific topics emerged, the Constitution was finally promulgated in December 1947 and came into force in January 1948. According to Viroli (2012, p. 67), “it was born from the pursuit of the compromise—with a positive meaning—, namely from the will to define a text that could be accepted by all components of the Italian society, beyond the diverse interests and the diverse cultural tendencies.”^6^

The Italian Constitution is composed of three sections: “Fundamental Principles”, “Part I. Rights and Duties of Citizens”, “Part II. Organization of the Republic”.^7^ Here, we will focus our analysis on the first two sections because those are more relevant to the purposes of this work, as the third section concerns the organs of the Republic (Parliament, President, Government, Judicial Branch, Local organs, and others). Since the Constitution’s promulgation, although the third section has undergone some important revisions, there has never been any significant appeal or attempt to change the Fundamental Principles and Part I. This may be interpreted as indicative of a broad and persistent support for these sections among the Italian public.

For all the above reasons, Italy is a suitable case study for the application of the Constitutional Approach for the selection of dimensions. It is important to stress that this is not an isolated case; there are, indeed, many other countries where this approach could be applied, such as Spain, Belgium, Greece and Luxemburg in Europe. Outside Europe, the constitutions of Mexico, Costa Rica, Brazil, South Africa, Namibia, and India, for example, seem particularly promising as highlighted by Burchi et al. (2015). More research, however, is needed in this direction.

Considering the cultural and social circumstances of the 1940s, it is no surprise that the Italian Constitution never mentions the word “well-being” or its synonyms. On the contrary, the concept of poverty is mentioned—although only once—in Article 32, which states: “The Republic … guarantees free medical care to the indigent.” We show that the absence of any literal reference to well-being does not mean that the “Constituent Fathers” were not concerned about it. In fact, although a comprehensive and many-sided concept of well-being is not explicitly conceived in the Constitution, it is possible to find many clear and significant references to a number of fundamental well-being dimensions, labeled as “rights” and/or “freedoms”. This is consistent with the capability approach, as capability “can be read as a reflection of substantive freedom” (Sen 1992, p. 49).

With reference to Sen’s concepts of well-being and agency recalled in Sect. 1, we will consider only those rights and freedoms that are directly and specifically related to *well*-*being* achievement and *well*-*being* freedom. Consequently, those rights and freedoms that are related to *agency* rather than *well*-*being* will be not included in order to keep a consistent theoretical framework.

Before detecting well-being and poverty dimensions in the text of the Constitution, one cannot but notice an extraordinary affinity between one of the fundamental principles of the Constitution and the “Human Development Paradigm”, which is largely based on the capability approach. The second paragraph of Article 3 refers to the “full development of the human person”. According to Giorgio La Pira,^8^ one of the most authoritative fathers of the Constitution, this is the ideal to which the Constitution aimed. Likewise, “human development … is about creating an environment in which people can develop their full potential” (UNDP 2001, p. 9). Therefore, the Italian Constitution and the Human Development Paradigm, although crafted in very different times and milieus, have a common foundation.

Starting with the Fundamental Principles (Arts. 1–12), it is very significant that the first article of the Constitution states “Italy is a Democratic Republic, founded on work”. Hence, the first well-being dimension that is cited is “work”. Shortly after, Article 4 (“… the right of all citizens to work …”) reinforces this priority.

A number of other articles are, then, dedicated to this dimension. In Sect. 2, on the “Rights and Duties of Citizens”, there is a subsection on “Economic Rights and Duties”, which includes as many as six articles about work: Article 35 (“The Republic protects work …”), Article 36 (Work remuneration and hours), Article 37 (Work of women and minors), Article 38 (Social security and protection), Article 39 (Free trade unions), Article 40 (The right to strike). Given the position of “work” in the text (Art. 1) and the numerous articles dedicated to it, there are good grounds to argue that this is the most valued dimension of well-being in the Italian Constitution. This is not a surprise, given the important role of socialists, communists and social Christians in the Constituent assembly. Furthermore, considering the protections and safeguards provided by the above mentioned articles, it is not just “work” per se, that is considered valuable, but rather “decent work”, to use a concept coined by ILO (1999).

Two other fundamental poverty dimensions can be detected in Sect. 2, in the subsection on “Ethical and social rights and duties”:

- Article 32 is about “health” and “medical care”, and is the only one about this dimension;
- Articles 33 and 34 are about “education”. Also Article 35, dedicated to work, cites one important aspect of education: “The Republic … provides for the [vocational] training and professional advancement of workers”.

In Article 9 of section 1, we find another relevant well-being dimension: “the development of culture and … research” and the safeguard of “the historical and artistic heritage”. Although citizens’ access to or enjoyment of culture and arts are not explicitly cited, there is in the Constitution a certain attention to the cultural dimension of well-being.

A further group of articles is about rights that concern “participation”, in its various forms:

- In section 1, Article 3: “actual participation of all workers in the political, economic and social organization of the country”^9^;
- *Civil participation*, in section 2, subsection I (Title I. Civil rights and duties): Article 17 on “the right to assemble”, Article 18 on “the right to form associations”, Article 21 on people’s “right to express freely their ideas” (including press);
- *Economic participation*, in section 2, subsection III (Title III. Economic rights and duties): Article 41 on free private economic initiative, Article 45 on co-operative enterprises and artisanal work, Article 46 on workers’ collaboration in the management of enterprises;
- *Political participation*, in section 2, subsection IV Political rights and duties: Article 48 on the right and duty to vote, Article 49 on free association in parties, Article 50 on citizens’ petitions; Article 51 on citizens’ eligibility.

From these articles it emerges that civil, economic and political participation receive much attention, similar to that given to “work”. Also, a strong connection exists between the latter and economic participation.

Regarding poverty, besides the above-mentioned Article 32, which considers medical care as a basic entitlement, an indirect reference is made also in articles 117 and 120, which are in Part II of the Constitution. Those two articles were modified in 2001 by a constitutional law that re-defined the relations between the central and the local governments. We will refer to that latest version.

In the long new Article 117, regarding the subject matters on which the central government has exclusive powers, one of the subjects is the “determination of the basic level of benefits relating to civil and social entitlements to be guaranteed throughout the national territory”. Furthermore, Article 120 says that “The [central] Government can act for [local governments] … whenever such action is necessary … to guarantee the basic level of benefits relating to civil and social entitlements”.

Both articles 117 and 120 recall the idea of a “basic level of benefits relating to civil and social entitlements to be guaranteed”, i.e. a basic level to which any citizen is entitled and below which she risks becoming poor. Using the terminology of the capability approach, we may say that the Italian Constitution guarantees to any citizen—at least in principle—a minimum level of basic “functionings”. Notwithstanding the declared concern for a basic level of benefits that may avoid poverty, in the Constitution—apart from “health” cited in Article 32—no specific poverty dimensions are explicitly mentioned. Therefore, we may assume that the “basic level of benefits … to be guaranteed” refers to the “civil and social entitlements” that we have detected in the first two sections of the Constitution.

This short review shows that at least seven fundamental well-being and poverty dimensions are clearly embedded as public ideals in the Constitution. According to their position and to the space respectively dedicated to them in the document, we may order them in the following way: (1) decent work; (2) political, (3) civil and (4) economic participation; (5) education; (6) health; (7) culture, arts and science.

May we consider those seven dimensions valid also for poverty? Taking together the concept of poverty that we have adopted in Sect. 1 and the text of the Constitution, the first six dimensions may be regarded as poverty dimensions, too.

It is also evident that a number of important dimensions, which may be found in the vast literature on poverty and well-being or in other constitutions, are missing. Just to give some significant examples of missing dimensions, let us consider the OECD Better Life initiative (OECD 2011) and the Italian Equitable and Sustainable Well-Being (BES) (Cnel and Istat 2013). Both include “environment” (i.e., living in a clean and safe environment), “safety”, and “community/social relationship”; the OECD also includes “housing”. The last two dimensions are particularly relevant for escaping poverty.

It is difficult to understand why there is no reference to such important dimensions in the Italian Constitution. Of course, one reason could be the fact that it dates from 1947. This may, for instance, explain the absence of the environmental dimension, as in the 1940s—especially in Italy—there was much less awareness about ecological problems than there is today. However, this cannot explain the absence of the “housing” dimension, which has always been a major problem in contemporary Italy and was officially considered as a fundamental “dimension of poverty” (Braghin 1978, ch. 2) at the beginning of the 1950s. Possibly, ensuring full and decent employment to all workers was then considered a sufficient condition for achieving a number of other basic functionings, such as housing and social inclusion (which is related to “community/social relationships”). Recent extensive evidence of working poor—and even of working homeless (Navarro 2013)—suggests that work alone is not a sufficient condition to ensure decent housing or social inclusion, because of low-wage jobs.

The lack of any explicit reference to some important dimensions should not be considered a reason for rejecting the Italian Constitution as an appropriate informational base for choosing the relevant poverty and well-being dimensions. In fact, as Sen writes, “the problem of valuation is not … one of an all-or-nothing kind” (1999, p. 78): The incompleteness—also typical of the capability approach—should not be a source of embarrassment. Therefore, we can utilize the seven dimensions from the Constitution pointed out above as a core, minimum list, or as a starting, open-ended list of fundamental dimensions, which may be integrated—if and when it is necessary—with further ones selected according to analogous, equally consistent criteria.

From this perspective, there are at least four different possible sources of integration. The first are the dispositions of the Constitutional Court, established in 1956, which is in charge of “ensuring the constitutionality of laws, with special attention to the protection of fundamental rights, and of mediating social conflicts, thereby contributing essentially to the adaptation of the legal system to the evolution of society.”^10^ Since that moment, the Court has identified “new” social rights, i.e. rights that are not explicitly codified in the Constitution but are implicit in it because they are in line with the fundamental principles, such as the “full development of the human person” or the principle of equality.

The first important example is the right to a decent housing. While this was absent from the Constitution, since the second half of the 1980s the Court has gradually made some judgments that have given birth to this new right. It is, in particular, Decision 217 of 1988 that marks a change: here the Court declares that “The right to housing represents one of the fundamental characters of the democratic State designed by the Constitution. The task to create the minimum conditions of a social State and guarantee to the highest possible number of citizens a fundamental social right, like the one to housing, contributes to the achievement of a higher level of human dignity and cannot be, in any way, renounced by the State”. All the following judgments of the Court on this topic confirmed and reinforced the existence of this right. Examples are Decision 119 of 1999, where it states that the “right to a decent housing is undeniably comprised within the fundamental rights of the person” and Decision 520 of 2000 that concerns social housing to guarantee the “access of less affluent classes to the rental market by contributing to the costs related to the payment of rent”.

In the same period, a series of Court judgments introduced the right to a healthy environment. The milestone is Decision 641 of 1987, where the Court states “The environment is protected as a determinant element of quality of life. Its protection does not pursue naturalistic or aesthetic abstract purposes, but it expresses the need for a natural habitat in which man lives and acts, and which is necessary to the community and, for it, to the citizens, according to values widely shared; it is firstly due by constitutional precepts (Arts. 9 and 32 of the Constitution), so that it becomes a primary and absolute value”. Therefore, it recognizes the fundamental right to a healthy environment under Art. 9 (“[The Republic] shall safeguard the natural landscape and the historical and artistic heritage of the Nation”) and Art. 32 (“The Republic shall safeguard health as a fundamental right of the individual and as a collective interest”). In line with our “political construction” of well-being dimensions, it is important to include “decent housing” and “living in a healthy environment”.^11^

A second source for integrating our list of dimensions is the European Union legislation, as Italy has taken part in the European integration process since the first stage. As already shown by existing jurisprudence and case law, it is legitimate to refer to European treaties for subjects not explicitly considered in the Italian Constitution.

A third possible source is international treaties. As the Constitution recognizes in various articles the role of international treaties, conventions and agreements, one can refer to treaties ratified by Italy as an additional source of relevant well-being dimensions.

The last, but not least, source derives from Art. 2 (initially proposed by Giorgio La Pira in 1947): “The Republic recognizes and guarantees the inviolable rights of the person”. Although the Universal Declaration of Human Rights is not cited, because it was adopted by the UN General Assembly on December 10, 1948—just 1 year after the Italian Constitution—the reference to the “inviolable rights of the person” made by Art. 2 permits us to consider also that Declaration as a possible source of valuable well-being dimensions for Italy.

An approach that integrates the Constitution with such aforementioned sources may be defined as an “Enlarged Constitutional Approach”. Such an approach is preferable to an approach based *only* on the Constitution, because it allows for a consideration of official interpretations and extensions of the constitution, which makes it alive and up to date. It becomes increasingly necessary as a constitution ages.^12^

Finally, we argue that our Enlarged Constitutional Approach satisfies the criteria proposed by Ingrid Robeyns (2005) to choose dimensions. Our list is, in fact: (1) explicitly formulated, justified and should be viewed as an initial list, open to further discussion; (2) based on a methodology that has been justified on ethical grounds and can generally work for the objective of measuring well-being (and poverty) in Italy (see next section); (3) “ideal”, which can at a later stage be changed into a feasible one, depending on data availability (see next section for an example); (4) exhaustive and not further reducible.

## An Empirical Application

In this section, we look at the implications of using the set of dimensions extrapolated from the Constitution. In particular, we compare well-being levels in Italian regions obtained using the dimensions highlighted in Sect. 4 with those obtained in a recent initiative launched by the Italian National Institute of Statistics (Istat), together with the National Council for Economy and Labor (Cnel). The objective of this initiative was to measure the Equitable and Sustainable Well-being (BES) in the country (Cnel and Istat 2011).

As highlighted in Sect. 4, we derived seven well-being dimensions from the Italian Constitution. Unfortunately, we did not find regional data for suitable indicators of civil participation: therefore, we had to remove this dimension from our analysis. This is a typical example of constraints that researchers face in the attempt to operationalize an ideal list.

In Table 1 we report the list of six remaining dimensions. We initially assign equal weights to all the dimensions, but in a second stage we also propose a preliminary, alternative weighting scheme, based on three main factors: (a) number of articles that refer to these dimensions; (b) space dedicated to the dimensions (as articles may have very different size); (c) articles’ position in the Constitution, as the opening articles are deemed more important than subsequent ones. Since we did not find a way of assigning weights depending on attributes (b) and (c), we relied dominantly on attribute a). As about 4/5 of article 35 refers to decent work, while only 1/5 to education, we attributed 0.8 articles to the former and 0.2 to the latter. In the case of health and culture we assigned the same weight not just because 1 article is dedicated to each of these dimensions but also because the article (no. 9) dedicated to culture comes before that on health (no. 32) but is shorter. Finally, we notice a correlation between the three attributes in the other dimensions: for example, the weight assigned to “decent work” (0.378) through frequency of reference, ended up much larger than that for the other dimensions, and decent work is the dimension cited earliest in the Constitution, in Articles 1 and 3.

**Table 1:** List of dimensions and weights based on the constitutional approach

Ranking	Constitutional approach			Enlarged constitutional approach		
	Dimension	Article numbers	Weight	Dimension	Article numbers	Weight
1	Decent work	6.8	0.378	Decent work	6.8	0.340
2	Political participation	4	0.222	Political participation	4	0.200
3	Economic participation	3	0.167	Economic participation	3	0.150
4	Education	2.2	0.122	Education	2.2	0.110
5	Health and medical care	1	0.056	Health and medical care	1	0.050
5	Culture and arts	1	0.056	Culture and arts	1	0.050
				**Decent housing**	**1**	0.050
				**Environment**	**1**	0.050

We then consider also an Enlarged Constitutional Approach, in which we include the dimensions that result from the recent Constitutional Court’s interpretation and actualization of the Constitution, namely “living in a decent house” and “living in a healthy environment”. Assuming that a Constitution written in the 21st century would dedicate one article each for the two new dimensions, we applied a new set of weights (see last column of Table 1).

Moving to the approach used by Cnel and Istat to generate the BES, these institutions have made an initial “proposal for domains”, which consisted of 12 dimensions.^13^ Then, they elaborated a short online questionnaire and submitted it to a (non-representative) sample of Italian citizens in 2011–2012. The questionnaire intended to verify whether people were satisfied with the existing way of measuring well-being and, above all, whether they considered “very important” or “of little importance” each of the 12 dimensions initially proposed.^14^

This method can be classified as an “empirical survey” approach, explained in Sect. 2. In the first three columns of Table 1 we report the ranking of each dimension, the name of the dimension and the percentage of population who considered that specific dimension “very important”. We can see that health is the most important dimension, followed by the environment and education. Based on people’s answers, we constructed a straightforward set of weights (column 4): this ranges from 0.104 for health (the most valued dimension) to 0.06 for economic well-being (the least-valued dimension).^15^

In order to ensure a better comparison with the Constitutional approach, we also considered a shorter list of the BES dimensions. In the second and third panel of Table 2 we report the new weights with 6 and 8 dimensions, respectively.

**Table 2:** List of dimensions and weights based on the BES/empirical survey approach

Rank	Full BES (12 dimensions)			BES-6 dimensions		BES-8 dimensions	
	Dimension	% very important	Weight	Dimension	Weight	Dimension	Weight
1	Health	98.00	0.104	Health	0.179	Health	0.140
2	Environment	95.10	0.101	Environment	0.173	Environment	0.135
3	Education & training	92.40	0.098	Education & training	0.169	Education & training	0.132
4	Quality of services	91.20	0.096	Quality of services	0.166	Quality of services	0.130
5	Work & life-work bal.	88.80	0.094	Work & life-work bal.	0.162	Work & life-work bal.	0.126
6	Research & innovation	82.80	0.088	Research & innovation	0.151	Research & innovation	0.118
7	Culture and arts	77.80	0.082			Culture and arts	0.111
8	Social relations	76.30	0.081			Social relations	0.109
9	Safety	69.70	0.074				
10	Political part./trust in inst.	62.80	0.066				
11	Life satisfaction	55.70	0.059				
12	Economic well-being	55.20	0.058				

After a consultation process, Cnel and Istat elaborated a long list of indicators to reflect the different dimensions.^16^ We mostly drew from this list to generate composite, multidimensional, indicators of well-being for Italian regions. In particular, wherever possible we chose “outcome indicators” (Burchi and De Muro 2016). Our full list of variables is reported in Annex 1. In the case of dimensions with two or more indicators, we aggregated them through geometric mean: the same function was used to finally aggregate all the dimensional indicators. This procedure, adopted also for the new Human Development Index (UNDP 2010), accounts for imperfect substitutability across dimensions.

Before starting this exercise, it is important to clarify that Istat had no intention of building a composite indicator of well-being. Therefore, the following elaborations are only conducted to illustrate an example of the implications of using the Constitutional Approach.

**Fig. 1:**
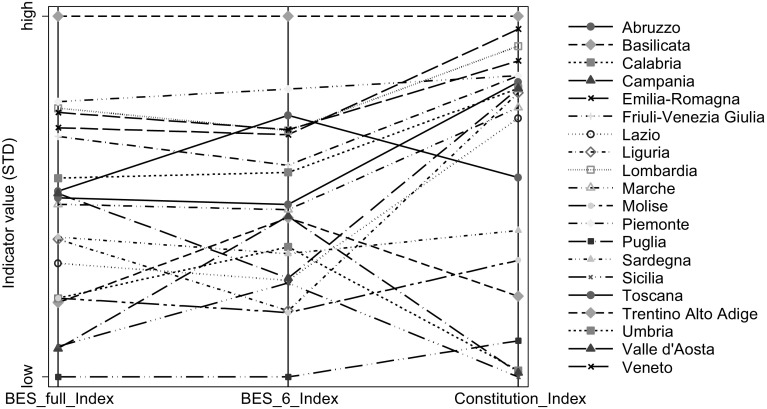
*Parallel plot* with the comparison between the BES-type Index (full and with 6 dimensions) and the Constitution-based Index (dimensions carry equal weights)

Paraphrasing a highly cited paper on poverty (Ruggeri-Laderchi et al. 2003), does it matter that we don’t agree on the list of well-being dimensions? In Fig. 1 we report the parallel plot of three indices: (1) a full BES-type Index (12 dimensions); (2) a BES-type Index with 6 dimensions, (3) the Constitution-based Index. A problem in the direct comparison between indicators (1) and (3) could be that the different *number* of dimensions (12 vs. 6) could be more important than the different type of dimensions in explaining the different rankings of Italian regions.^17^ Therefore, we introduce indicator (2), which includes the same number of dimensions as indicator (3). In this first stage, we assign equal weights to the dimensions.

One of the main strengths of the parallel plots consists in the possibility of visualizing both the changes in relative (standardized) values and rankings in the different regions due to a change in the composite index. As results in terms of ranking are striking, here we concentrate on them. The first point we can make, looking at Fig. 1, is that, independently of which indicator we use, regions from South Italy always have the worst values, while regions from the Center and the North have higher values and more similar scores. In particular, Trentino Alto Adige ranks 1st according to all indicators. However, the choice of indices does make a substantial difference within these macro groups.

The most evident case is the Southern region of Abruzzo, which, relatively speaking, performs much better in the BES-type Index. This region loses 5 positions if we use the set of dimensions directly derived from the Constitution instead of the 12 included in the BES. Calabria (Southern region) and Piemonte (Northern region) also lose a considerable number of positions: 3. On the other hand, two Northern regions (Liguria and Emilia-Romagna), one region in the Center (Toscana) and one from the South (Puglia) gain 3 positions as a result of the same shift.^18^

If we then compare the Constitution-based Index with the reduced BES-type index we notice that the variation in regions’ positions is even more striking. Abruzzo, for example, now loses 10 positions.

**Fig. 2:**
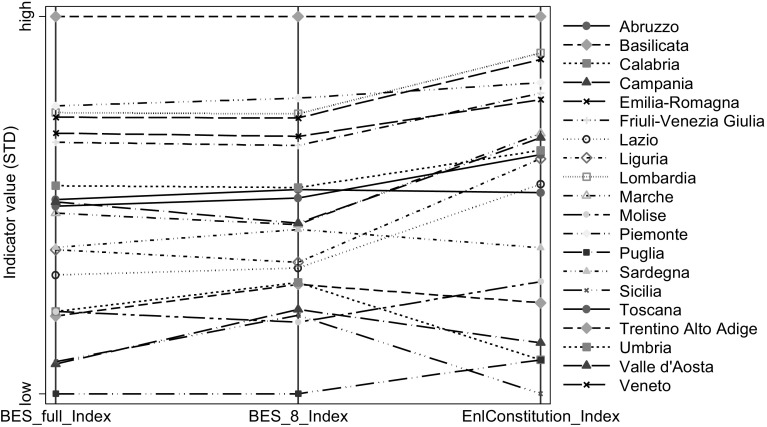
*Parallel plot* with the comparison between the BES-type Index (full and with 8 dimensions) and the Enlarged Constitution-based Index (dimensions carry equal weights)

In Fig. 2 we compare the regions’ performances on the basis of the Enlarged Constitution-based Index with the full BES index and the one with only 8 dimensions. The general picture is the same as the one emerging from Fig. 1: a large group of countries changes position. In particular, Abruzzo’s and Calabria’s rankings fall from 8th to 13th position and from 15th to 19th position, respectively, when we measure well-being using the extended list of Constitutional dimensions instead of the BES dimensions. Very similar findings are obtained when the full BES index is replaced with the one with only the first 8 dimensions.

**Fig. 3:**
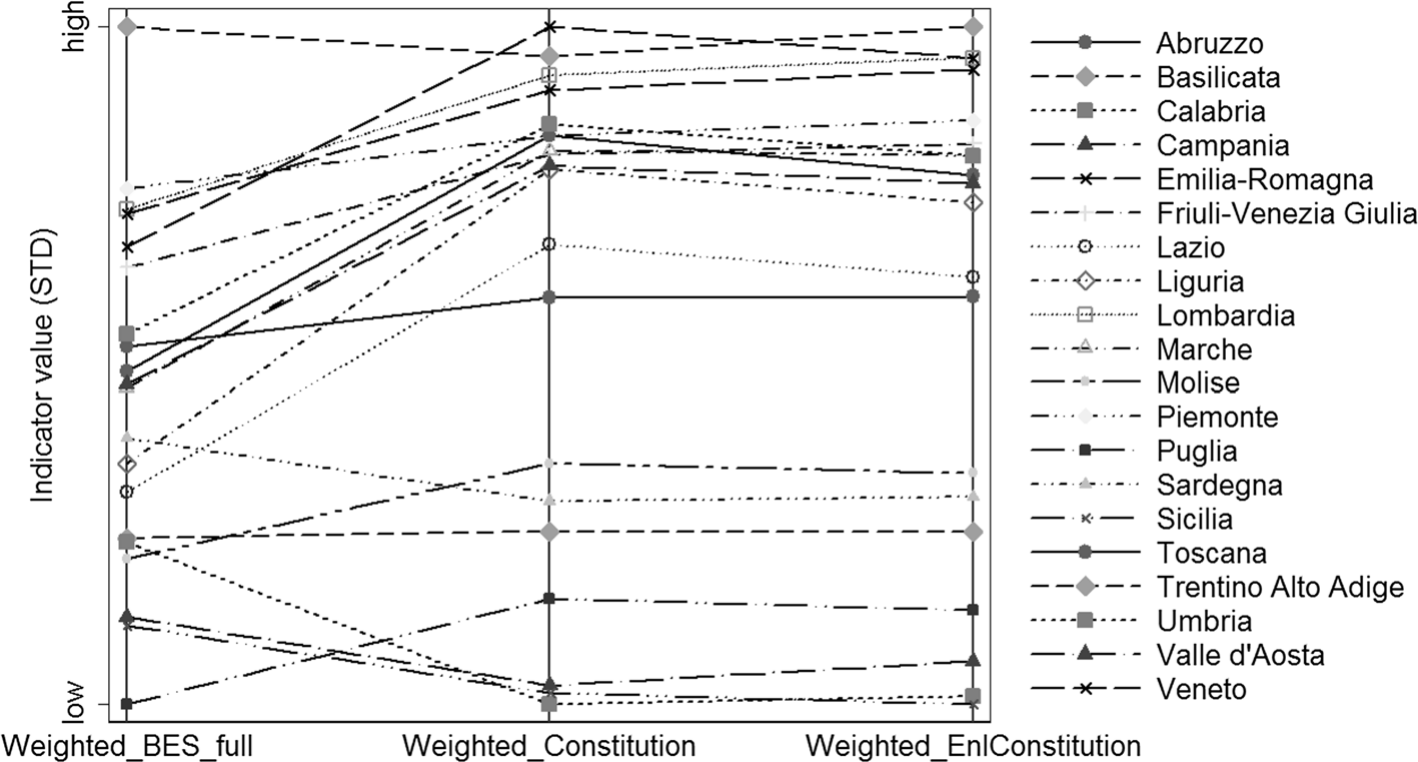
*Parallel plot* with the comparison between the full BES-type Index, the Constitution-based Index and the Enlarged Constitution-based Index (alternative weighting schemes)

Finally, we analyzed the same relationship using the weighting schemes presented in Tables 1 and 2. The correlation is about the same as the one with equal weights (Fig. 3). When adopting the Constitutional Approach, the ranking of Abruzzo and Piemonte worsens by 5 positions, while that of Calabria by 4: in contrast, Emilia Romagna gains 4 positions, while Marche, Molise, Puglia and Toscana, 3. Results look very similar with the Enlarged Constitution-based Index.

We therefore argue, from this empirical analysis, that the choice of dimensions is not only relevant because a list of dimensions can be more or less “legitimate” on the political ground and thus more likely to be endorsed by society and policy-makers. It also matters because, depending on the method we use, we obtain different values of well-being indicators and even different rankings among the geographical units analyzed. This has direct implications for the identification of geographical and horizontal inequalities and for the targeting of policy interventions.

## Concluding Remarks

Empirical papers examining multidimensional well-being or poverty often pay little attention to a crucial step in the measurement exercise: the selection of relevant dimensions. Data availability is the dominant criterion guiding this choice. In the present paper we argue in favor of a Constitutional Approach, using the constitution as a starting point for a discussion on well-being or poverty dimensions. This method can be considered as a revised version of the public consensus approach.

We tested this method in the case of Italy, where a long-standing Constitution enjoys wide consensus. Through a “textual” analysis of the Constitution we derived 7 main dimensions. However, we argue for an Enlarged Constitutional Approach, i.e. a broader interpretation of the “Constitution”. Looking at the judgments of the Constitutional Court we detected two further fundamental dimensions not explicitly codified in the original document, i.e. “living in a decent house” and “living in a healthy environment”.

A preliminary empirical exercise shows that the adoption of this approach not only provides us with an ethically justifiable set of public values, and therefore is better than other approaches, but also leads to different results. The comparison of the rankings of Italian regions on composite well-being indices calculated using the Constitution-based dimensions and the dimensions utilized in the BES initiative reveals important differences: a number of regions perform differently.

While in this paper we applied our approach to the Italian Constitution, this is neither limited only to nation states nor to countries with written constitutions. It is important to keep in mind that the list arrived at through interpreting a specific Constitution at are not meant as universal principles. They are meant to apply to the context under examination; in our case Italian measurement of well-being/poverty and policy-making. However, similar exercises may be carried out in different contexts (EU, International Institutions), by taking different public doctrines as starting point. Moreover, in contexts such as those of some countries, where formal, written constitutions do not exist, public ideals may be detected, for example, in oral traditions of fundamental values. What remains an important question for further inquiry is how to draw the moral limits of suitable constitutions. Constitutions are very different, some more adequate than others, and some involve public ideals that are deemed morally dubious. We have offered some examples of procedural and substantive criteria to distinguish good and bad constitutions in this respect, but a full set of conditions is subject to further research.

In conclusion, we believe that this new method of selecting dimensions can provide important and original insights for the construction of theoretically sound, politically legitimate indicators of well-being and poverty. Where the basic conditions are satisfied, this method also offers a viable and pragmatic solution as it does not require the collection of additional data. While the present paper focuses on a high-income country, Italy, it can be employed in low-income countries, too. Clearly, this exercise may be more straightforward in the case of long-standing democracies, such as India.

## Appendix

See Table 3.

**Table 3:** List of indicators

Composite index	Dimension	Indicators
BES & Constitution (2)	Health	Female life expectancy at birth
		Male life expectancy at birth
BES & Constitution (2)	Knowledge	Proportion of population with higher education
		Proportion of population with university degree
		(1−) proportion NEET
BES & Constitution (2)	Decent work	Employment rate (20–64 year)
		Share of employed persons in regular occupation (proportion of total employed persons)
		Average work satisfaction (0–10 scale)
Enlarged Constitution	Decent Housing	(1−) proportion of families declaring that their house is too small
		(1−) proportion of families declaring that their house is in bad conditions
BES & Constitution (2)	Political participation	Voter turnout: percentage of eligible voter who cast a ballot in the last election for the European Parliament
		Civic and political participation: aggregation of the following indicators: Share of people aged 14 and over who talks about politics at least once a week; Share of people aged 14 and over who seek information about Italian politics at least once a week; Share of people aged 14 and over who in the past 3 months have taken part in online consultations or polls on civic/political issues (e.g. urban planning, signing a petition); Share of people aged 14 and over who in the past 3 months have read and posted on the web opinions on social/political issues
Constitution (2)	Economic participation	Activity rate: active population as a proportion of total population of working age
BES	Social relations	Satisfaction with family relationship: Share of population aged 14 and over who have declared to be very satisfied with its family relationships
		Satisfaction with friendship relationship: Share of population aged 14 and over who have declared to be very satisfied with the relationship with his/her friends
BES	Economic well-being	Proportion of population not at risk of (relative) poverty
		Proportion of population without severe material deprivations
BES	Safety	(1−) Homicide rate (Number of homicide/population * 100,000)
		(1−) Proportion of women who were victims of physical violence
BES	Life satisfaction	Life satisfaction index: proportion of people aged 14 and over with a level of life satisfaction from 8 to 10
BES & Enlarged Constitution	Environment	Emissions of CO_2_ and other greenhouse gasses: tonnes of CO_2_ equivalent per capita
		CO_2_ Equivalent per inhabitant. Normalized with respect to the maximum and minimum in the European Union)
		Urban waste collected (in Kg) per inhabitant. Normalized with respect to the maximum and minimum in the European Union)
		Share of collected waste on total waste
BES	Research and Innovation	Innovation rate of the national productive system: proportion of enterprises with (process, product, organizational or marketing) innovation on total enterprises with 10 or more employees
		Proportion of product innovators: proportion of enterprises with product innovation on total enterprises with 10 or more employees
BES & Constitution (2)	Culture and arts	Proportion of people aged 6 and over who read at least one book in the 12 months preceding the interview
		Proportion of people aged 6 and over who read the newspaper at least once a week in the 12 months preceding the interview
		Proportion of people aged 6 and over who, in the 12 months preceding the interview, have gone at least once to exhibitions and museums
		Proportion of people aged 6 and over who, in the 12 months preceding the interview, have gone at least once to archaeological sites, or monuments
BES	Quality of services	Composite index of service accessibility: proportion of individuals who find it very difficult to reach some basic services (pharmacies, emergency, post office, police, carabineers, municipal offices, crèches, nursery, primary and secondary school, markets and supermarkets). Standardized values
		(1−) Prison density per 100 places: proportion of prisoners in penal institutions on the total capacity of penal institutions

## Footnotes

1 Although most functionings refer to objective states and actions, like being employed or participating in the life of the community, the capability approach does not rule out the subjective aspects of well-being. In fact, being happy or satisfied may be rightly considered as one of the functionings. The substantial difference with the subjective approach to well-being is that while the former is concerned only with perceptions and feelings, the capability approach has a much broader informational basis that includes many other dimensions and therefore may avoid the bias of “adaptation and mental conditioning” (Sen 1999, pp. 62–63).

2 Alkire discusses the issue of dimensions-selection with regard to poverty: however, the same dimensions can be used for well-being measurement given the relationship between poverty and well-being (Sect. 1). These two exercises may differ only in as much as we think that it should be up to the poor themselves to identify the relevant poverty dimensions (Narayan et al. 2000).

3 For a detailed exposition of the method see Burchi et al. (2014).

4 In her later work there is a shift in Nussbaum’s method of justification to an overlapping consensus borrowed from Rawls’ political liberalism. The core capabilities are now justified because they could allegedly gain support from a variety of moral and religious comprehensive doctrines. This hope or claim of universal endorsement, however, receives insufficient support in Nussbaum’s work, and seems incompatible with her earlier method of ethical theorizing. The question that is left unanswered is: How do we move from moral entitlements to political entitlements? Why should we think that dimensions derived from a singular ethical theory would gain wide consensus from adherents of different ethical systems? Our aim here, however, is not to refute Nussbaum’s method of justification but to flag our departures, and try to motivate our claim that the outcome of a political constructivist justification has a stronger normative potential for wide public appeal. For a detailed critical discussion of Nussbaum’s methods of justification see Claassen and Düwell (2013).

5 Nussbaum (2007) has an extensive study in which she uses the capability approach as a normative test for the American Constitution to see how well it does in securing basic entitlements grounded in her core group of capabilities. She does not, however, use the Constitution in the justificatory argument for selecting well-being dimensions.

6 Authors’ translation from Italian.

7 In this article we refer to the official translation in English of the Italian Constitution (Camera dei Deputati 2007).

8 Interview given in the 1950s, broadcast by the Italian Public Television (RAI) on 30/06/2008. Available at http://www.rai.tv/dl/RaiTV/programmi/media/ContentItem-29950599-0443-46af-a1cb-2bd7f25b2b0f.html#p=.

9 This is our own translation of the text and not the official one as in the latter the Italian word *effettiva* was erroneously translated as *effective* rather than *actual*.

10 Source: http://www.cortecostituzionale.it/documenti/download/pdf/Cc_Checosa_2013_UK.pdf.

11 This is in line with the idea that in our Constitutional approach, like in the capability approach, the selection of valuable well-being and poverty dimensions is not done once and for all, but it should be done again and again as the Constitution is possibly changed and interpreted (e.g. by the Constitutional Court) over time. We are not claiming that the list of dimensions is fixed.

12 For instance, this is the case of the US Constitution, the oldest modern democratic constitution. Not only it should be considered together with the Declaration of Independence and The Bill of Rights (known collectively as the *Charters of Freedom*), but also with the Subsequent Amendments to the Constitution (Amendments 11–27) and the numerous Supreme Court interpretations of the Constitution.

13 For the list of domains and its justification, see: http://www.misuredelbenessere.it/fileadmin/upload/docPdf/Cnel_Istat_committee_BES_domains.pdf.

14 The questionnaire and the results (only in Italian) are available at: http://www.misuredelbenessere.it/fileadmin/upload/docPdf/Questionario_per_il_sondaggio_on_line_sul_sito_del_BES.pdf and http://www.misuredelbenessere.it/fileadmin/relazione-questionarioBES.pdf.

15 It is important to highlight that the general objective of this survey was to understand people’s opinion on the importance of the different dimensions: the use of survey results to assign weight, as we do here, therefore goes beyond its original scope. The fact that respondents had only two possible answers creates further problems in assigning weights. In the literature on other countries, there are examples of *ad-hoc* questionnaires used to constructing a weighting system for the different dimensions (Chiappero-Martinetti and Von Jacobi 2012).

16 See: http://www.misuredelbenessere.it/fileadmin/upload/docPdf/LISTA_INDICATORI_ENG.pdf.

17 In our view, this is not a major problem as both a different number and a different list of dimensions are the direct results of the choice of the method used to derive them. The number of dimensions is, therefore, endogenous and not exogenous to the choice of the method. However, given that this problem could be exacerbated by the use of the geometric mean as aggregation function, we also compare indicators (2) and (3) in the attempt to isolate the effect of the number of dimensions.

18 We are aware that further analysis is needed to verify the robustness of these results to the use of different indicators. However, this falls outside of the scope of our paper.
